# Automatic segmentation of the maxillary sinus on cone beam computed tomographic images with U-Net deep learning model

**DOI:** 10.1007/s00405-024-08870-z

**Published:** 2024-07-31

**Authors:** Busra Ozturk, Yavuz Selim Taspinar, Murat Koklu, Melek Tassoker

**Affiliations:** 1 Department of Dentomaxillofacial Radiology, Faculty of Dentistry, Necmettin Erbakan University, Meram, Konya, 42050 Turkey; 2 Doganhisar Vocational School, Selcuk University, Konya, 42930 Turkey; 3 Department of Computer Engineering, Selcuk University Alaaddin Keykubat Campus, Konya, 42075 Turkey

**Keywords:** Cone beam computed tomography, Maxillary sinus, Deep learning

## Abstract

**Background:**

Medical imaging segmentation is the use of image processing techniques to expand specific structures or areas in medical images. This technique is used to separate and display different textures or shapes in an image. The aim of this study is to develop a deep learning-based method to perform maxillary sinus segmentation using cone beam computed tomography (CBCT) images. The proposed segmentation method aims to provide better image guidance to surgeons and specialists by determining the boundaries of the maxillary sinus cavities. In this way, more accurate diagnoses can be made and surgical interventions can be performed more successfully.

**Methods:**

In the study, axial CBCT images of 100 patients (200 maxillary sinuses) were used. These images were marked to identify the maxillary sinus walls. The marked regions are masked for use in the maxillary sinus segmentation model. U-Net, one of the deep learning methods, was used for segmentation. The training process was carried out for 10 epochs and 100 iterations per epoch. The epoch and iteration numbers in which the model showed maximum success were determined using the early stopping method.

**Results:**

After the segmentation operations performed with the U-Net model trained using CBCT images, both visual and numerical results were obtained. In order to measure the performance of the U-Net model, IoU (Intersection over Union) and F1 Score metrics were used. As a result of the tests of the model, the IoU value was found to be 0.9275 and the F1 Score value was 0.9784.

**Conclusion:**

The U-Net model has shown high success in maxillary sinus segmentation. In this way, fast and highly accurate evaluations are possible, saving time by reducing the workload of clinicians and eliminating subjective errors.

## Introduction

The maxillary sinus is a pyramidal space located in the body of the maxilla [1]. It is present at birth and is considered to grow until the end of 18 years of age. It grows vertically, horizontally, and anteroposteriorly. The largest growth period occurs in the first 8 years and is reported to reach maximum values in all diameters and volumes at the end of the 16th year [2]. The dimensions vary across individuals, and there may be discrepancies in right-left measurements within the same individual. The use of magnetic resonance imaging (MRI) and computed tomography (CT) is considered the gold standard for volumetric assessment, but access to these devices can be limited [1]. Compared to CT, cone beam CT (CBCT) is preferred for maxillary sinus evaluation due to its lower cost, smaller size, and lower radiation dose [3]. On the other hand, two-dimensional panoramic radiographs are frequently utilized for their convenience and minimal radiation exposure, although they may pose challenges in interpretation due to superimposition in the maxillary sinus region [4].

Medical image segmentation is a fundamental process in image analysis that involves dividing a medical image into meaningful components. This may involve organs, tissues, lesions, or other structures of interest. The goal of segmentation is to isolate and identify these regions of interest (ROIs) with high accuracy. By segmenting an image, healthcare professionals can gain a deeper understanding of the underlying anatomy and pathology. Precise segmentation allows better identification of abnormalities and diseases. Properly segmented structures can be used to plan surgeries, radiation therapy, and other interventions. Segmentation enables measurement of volumes, shapes, and other characteristics of ROIs; these can be crucial for monitoring disease progression or treatment response. Essentially, medical image segmentation helps uncover the wealth of information hidden in medical scans, leading to more precise and effective healthcare.

In recent years, deep learning algorithms have been used in the automatic segmentation of medical images. It has been reported that U-Net, which is used for this purpose, can perform segmentation with high speed and accuracy in CT [5] and CBCT segmentation [6]. This network framework offers the benefit of not only accurately segmenting the desired feature target and efficiently processing and objectively evaluating medical images but also enhancing diagnostic accuracy through image analysis [7]. Accurate segmentation of the maxillary sinus is important for dentists, ENT specialists, maxillofacial surgeons and maxillofacial radiologists, especially in treatment and follow-up processes where sinus volume may change, such as implant placement, orthognathic surgery and sinus augmentation [8]. When a disease like a cyst or tumor develops in the maxilla, changes in the maxillary sinus are crucial for diagnosis. Additionally, evaluating the maxillary sinus is essential in sinus lift procedures during implant surgery to provide the alveolar bone height required for the implant [4]. Although the maxillary sinus is a cavity with well-defined borders, it is a difficult structure to segment because it is close to the tooth roots and nasal passages and is often seen with thickened mucosa [8]. Correct segmentation of the maxillary sinus depends on the experience of the clinician and manual segmentation is quite time consuming [9].

Deep learning studies related to maxillary sinus in the literature are divided into two parts. While some studies investigate the diagnostic success of maxillary sinus pathologies with deep learning algorithms [3, 5] the other part focuses on maxillary sinus segmentation [5, 8, 10]. CT [5], panoramic radiographs [11] and, CBCT [8, 10, 12] have been used to segment the maxillary sinus.

In clinical practice, making an accurate analysis in manual segmentation performed by experts is laborious and time-consuming because the maxillary sinus; it is connected to nasal structures as well as neighboring anatomical structures such as frontal, ethmoid and sphenoid sinuses, and a multifaceted examination is required. In addition, the results of the analysis may vary greatly depending on the knowledge and experience of the experts [13, 14]. The aim of this study is to determine the automatic segmentation of maxillary sinuses with U-Net deep learning model on CBCT images.

## Methods

### Sample and study design

The present study was approved by the Research Ethics Committee of Necmettin Erbakan University Dentistry Faculty (no.2023/310) and was conducted in accordance with ethical regulations and relevant guidelines. Between February 2019 and April 2023, CBCT images with 500 × 500 pixel resolution of a total of 100 patients taken for different diagnostic purposes at Necmettin Erbakan University Faculty of Dentistry, Department of Oral and Maxillofacial Radiology were retrospectively collected. CBCT images were acquired using Morita 3D Accuitomo 170 (J Morita MFG Corp., Kyoto, Japan, operating at 90 kVp and 5 mA, 17.5 s rotation time, voxel 0.25 mm, 140 m × 100 mm field of view, 360° data acquisition, and no additional filtering) and NewTom GiANO cone beam 3D imaging (distributed by Verona, Italy, operating at 90 kVp and 10 mA, 18 s rotation time, voxel 0.15 mm, 140 m × 100 mm field of view, 360° data acquisition, and no additional filtering). Each patient was positioned parallel to the ground and the median line was standardized according to the equipments’ procedure.

Some criteria were considered when selecting CBCT images. The inclusion criteria were as follows; patient’s age ≥ 18 years, images with field of view (FOV) size where the maxillary sinus volume can be completely measured and with optimal diagnostic capability. Only healthy maxillary sinuses were evaluated (excluding mucosal thickening up to 3 mm). The exclusion criteria were as follows; patients younger than 18 years old, patients with craniofacial syndroms which affecting the maxillary sinus, head and neck trauma or surgery, cleft lip-palate, patients with maxillary sinus and jaw pathologies which affecting the borders of maxillary sinus, and recordings with artifacts in which the maxillary sinus cannot be clearly measured.

### Manuel segmentation of CBCT images

**Fig. 1:**
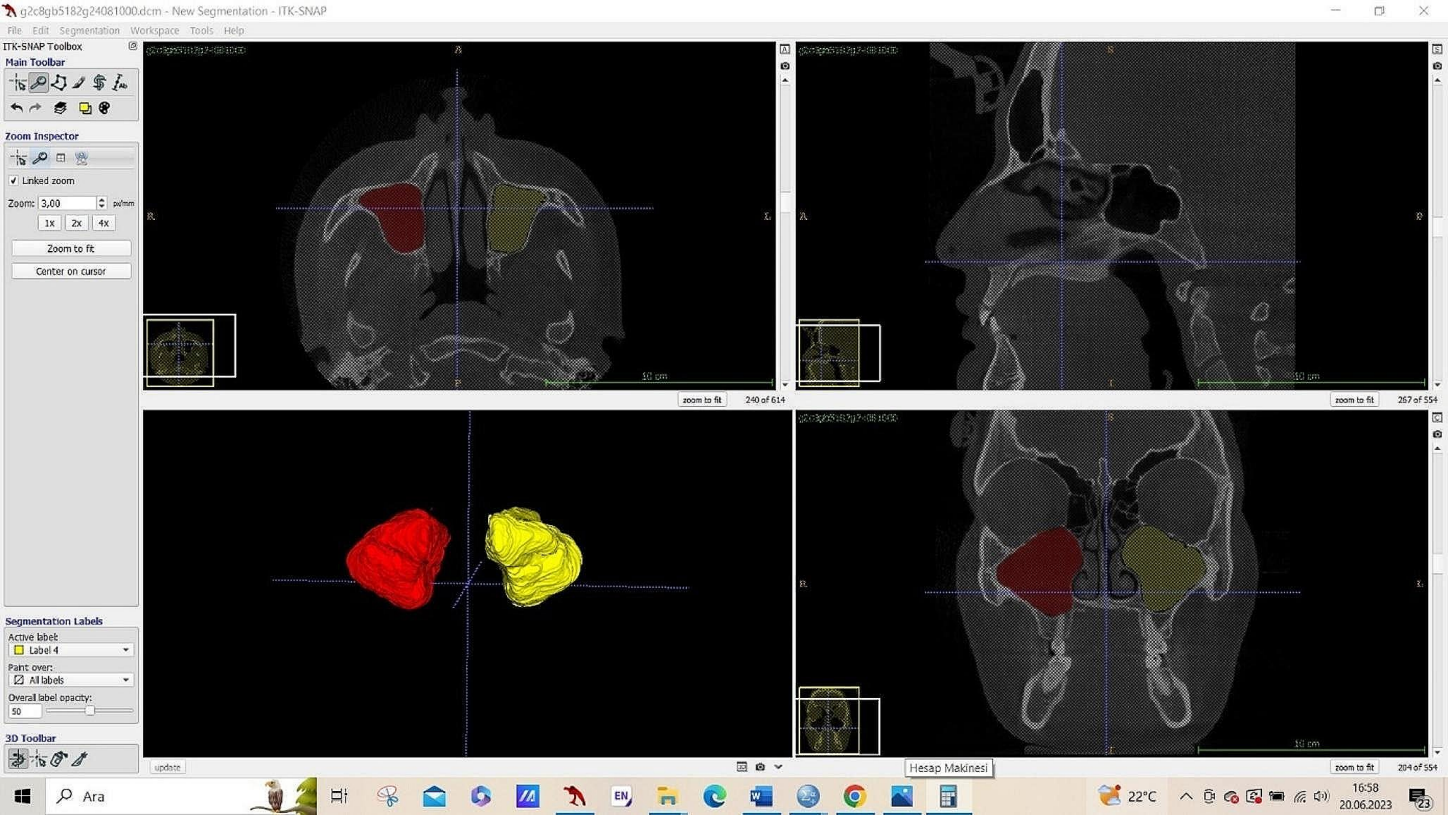
Marking of the maxillary sinuses in the ITK-SNAP software (version 3.8.0)

A total of 200 maxillary sinuses (right and left) were examined on axial sections. Maxillary sinus walls were marked by a physician with 12 years of experience in oral and maxillofacial radiology. For marking, ITK-SNAP (version 3.8.0) software was used (Fig. 1).

### U-Net

**Fig. 2:**
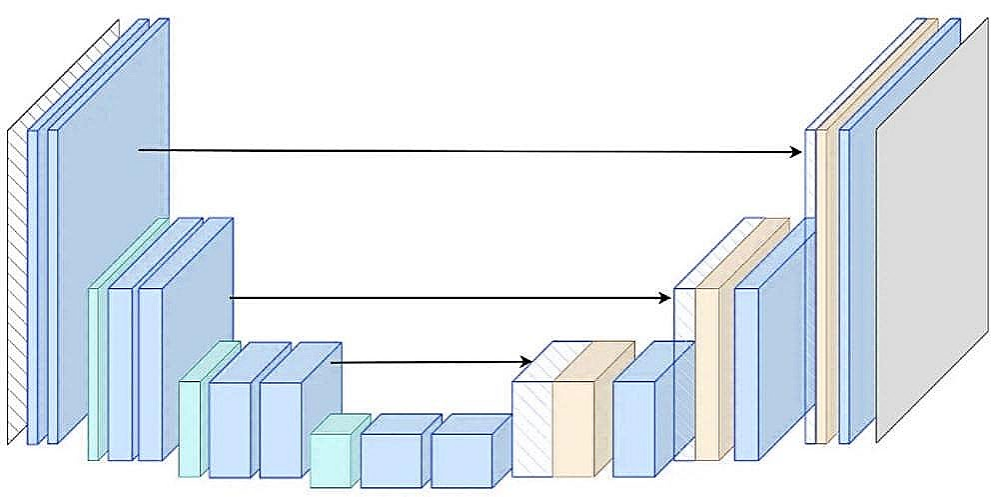
General representation of U-Net architecture

U-Net is a convolutional neural network (CNN) architecture that is a highly effective deep learning model in applications such as image segmentation and medical image learning. U-Net is particularly successful for illumination such as individual tumors or organ illumination, which is frequently used in medical image segmentation. Its name comes from the similarity of its structure to the letter U. The main features of the U-Net model are as follows: Deep Convolution Layers; U-Net consists of CNN layers. These layers are used to learn different properties of the ratios. The image progresses from lower-level layers that capture lower-level features to higher-level layers that capture higher-level features. Encoder and Decoder; U-Net consists of two main parts: Encoder and decoder. The encoder consists of transformed layers of a lower dimensional part of the input view. The code consists of layers in which these low-dimensional features of the solver are converted back to their original dimensions. Skip Links; an important feature of U-Net is that each layer in the encoder is linked to its corresponding decoding layer. These jump connections allow lower-level features to be combined with higher-level features. In this way, the model can better capture both local and global features and segmentation results can be sharper. Convolution and Concatenation (Convolution and Concatenation); after applying the convolution (convolution) process on the decoder layers, the features from the relevant encoder layer are combined. This prevents parts from getting lost. The U-Net model is successful in image segmentation tasks because it can segment at a high level of detail with both feature extraction and feature gap capability. This is quite useful in medical image processing, object differences, and other similar applications. U-Net can be adapted by developers and researchers as a basic model to more specific tasks. U-Net’s original model can be custom-solved to suit more specific applications and trained depending on the amount of data [15]. Figure 2 shows the general structure of the U-Net architecture.

The structure of the U-Net architecture used in the study is shown in Table 1.

**Table 1:** U-Net architecture used in the study

Layer information	Output	Number of parameters
input_1	(512, 512, 3)	0
conv2d	(512, 512, 64)	1792
spatial_dropout2d	(512, 512, 64)	0
conv2d_1	(512, 512, 64)	36,928
max_pooling2d	(256, 256, 64)	0
conv2d_2	(256, 256, 128)	73,856
spatial_dropout2d_1	(256, 256, 128)	0
conv2d_3	(256, 256, 128)	147,584
max_pooling2d_1	(128, 128, 128)	0
conv2d_4	(128, 128, 256)	295,168
spatial_dropout2d_2	(128, 128, 256)	0
conv2d_5	(128, 128, 256)	590,080
max_pooling2d_2	(64, 64, 256)	0
conv2d_6	(64, 64, 512)	1,180,160
spatial_dropout2d_3	(64, 64, 512)	0
conv2d_7	(64, 64, 512)	2,359,808
max_pooling2d_3	(32, 32, 512)	0
conv2d_8	(32, 32, 1024)	4,719,616
spatial_dropout2d_4	(32, 32, 1024)	0
conv2d_9	(32, 32, 1024)	9,438,208
conv2d_transpose	(64, 64, 512)	2,097,664
concatenate	(64, 64, 1024)	0
conv2d_10	(64, 64, 512)	4,719,104
conv2d_11	(64, 64, 512)	2,359,808
conv2d_transpose_1	(128, 128, 256)	524,544
concatenate_1	(128, 128, 512)	0
conv2d_12	(128, 128, 256)	1,179,904
conv2d_13	(128, 128, 256)	590,080
conv2d_transpose_2	(256, 256, 128)	131,200
concatenate_2	(256, 256, 256)	0
conv2d_14	(256, 256, 128)	295,040
conv2d_15	(256, 256, 128)	147,584
conv2d_transpose_3	(512, 512, 64)	32,832
concatenate_3	(512, 512, 128)	0
conv2d_16	(512, 512, 64)	73,792
conv2d_17	(512, 512, 64)	36,928
conv2d_18	(512, 512, 1)	65

The parameters of the data augmentation process, which is performed to ensure better training of the model by increasing the images in the dataset and to prevent overfitting, are shown in Table 2.

**Table 2:** Augmentation parameters of images for U-Net model

U-Net augmentation parameters
rotation_range = 15
width_shift_range = 0.05
height_shift_range = 0.05
shear_range = 50
zoom_range = 0.2

### Performance metrics

#### IoU (Intersection over Union)

**Fig. 3:**
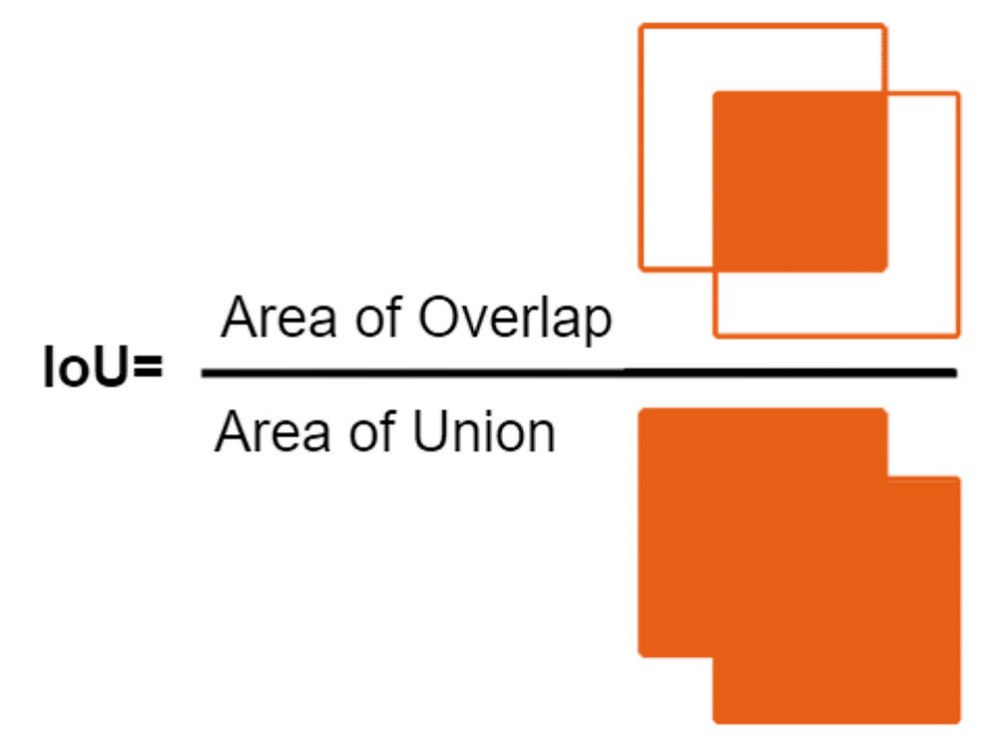
IoU calculation

IoU is an evaluation metric widely used in tasks such as image segmentation, object detail, and object recognition. IoU is used for how compatible two clusters can be. It is used to examine the accuracy of the object’s accurate object detail and object boundary. IoU is calculated as a value dividing the intersection of the following two sets by the union: IoU = (Intersection/Union). Intersection represents the designation of two objects or the joint representation of two parts. That is, it is the part of the model that is correctly detected and overlaps the real object or region. A union is the designation of two objects or the representation of the total division of two parts. It is the total area of the object detected by the model and the actual object or region. The IoU value usually varies between 0 and 1. In an ideal case, the IoU value would be close to 1 because the intersection covers the junction almost completely. The details of the model are very close. Lower IoU values indicate that the model’s detections are less consistent with real objects or region. IoU is used as an important metric, especially to evaluate object detection and segmentation evaluations. It is specifically used to examine the model through training and process and helps us better understand the sensitivity and accuracy of the model [16]. The calculation of IoU on the image is shown in Fig. 3.

#### F1-Score

F1-Score is an evaluation metric used especially in classification problems. F1-Score is a single number calculated by taking into account the precision and recall of a model. It is often used in classification problems to measure model performance on datasets with unbalanced class distribution. F1-Score is calculated as in Eq. 1.

1 $$F1-Score=2x\left(TP\right)/(2TP+FP+FN)$$

True positive (TP), False Positive (FP) and False Negatives (FN) are are the terms used in the calculation of F1 Score. F1-Score strikes a balance, taking into account both precision and sensitivity. Therefore, it is useful in classification problems with unbalanced data sets or where false positives and false negatives are equally important. F1-Score allows us to evaluate the model’s balance between true and false positives and false negatives. The F1-Score value varies between 0 and 1, and a higher F1-Score indicates better model performance. However, since F1-Score is a single number, using F1-Score alone may sometimes be insufficient to fully evaluate the performance of a classification model. Therefore, it is common to use it in conjunction with other metrics to evaluate a model’s performance in a broader context [17].

## Results

**Fig. 4:**
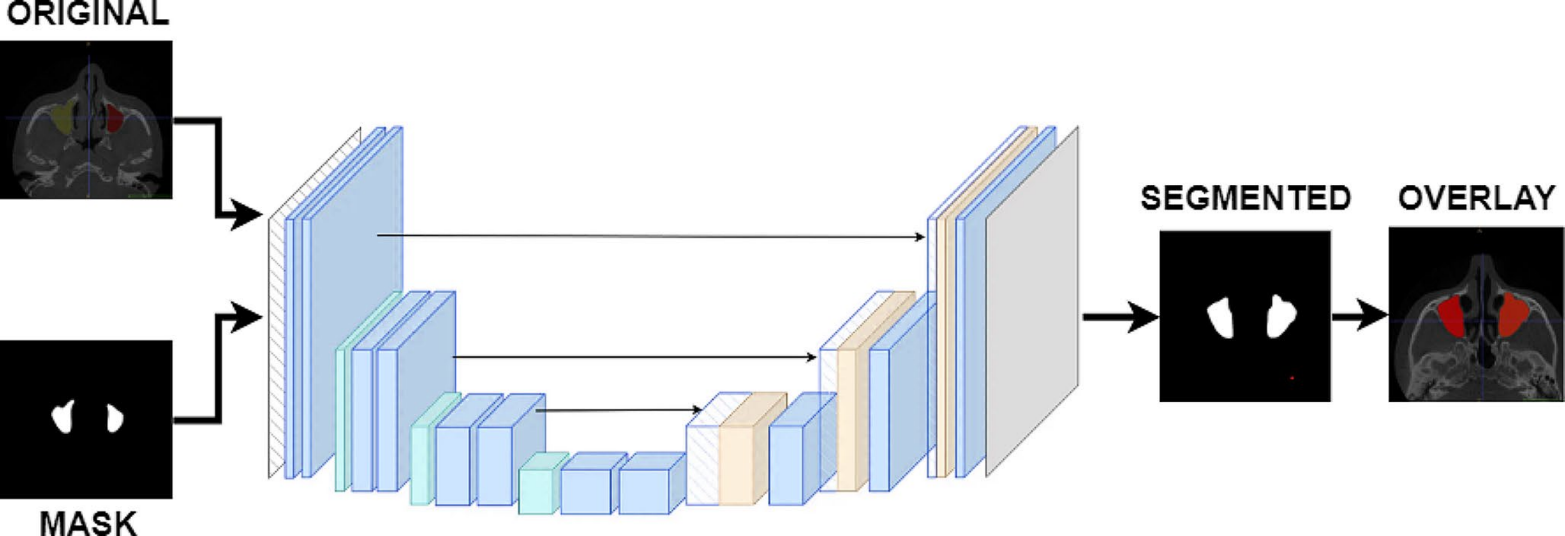
Flow chart of segmentation process

Masking operations were carried out so that the images in the dataset could be given as input to the U-Net model. The masked images and the original images were arranged in different folders with the same name and given as input to the U-Net model. The flow chart of the processes applied in the study is shown in Fig. 4.

**Fig. 5:**
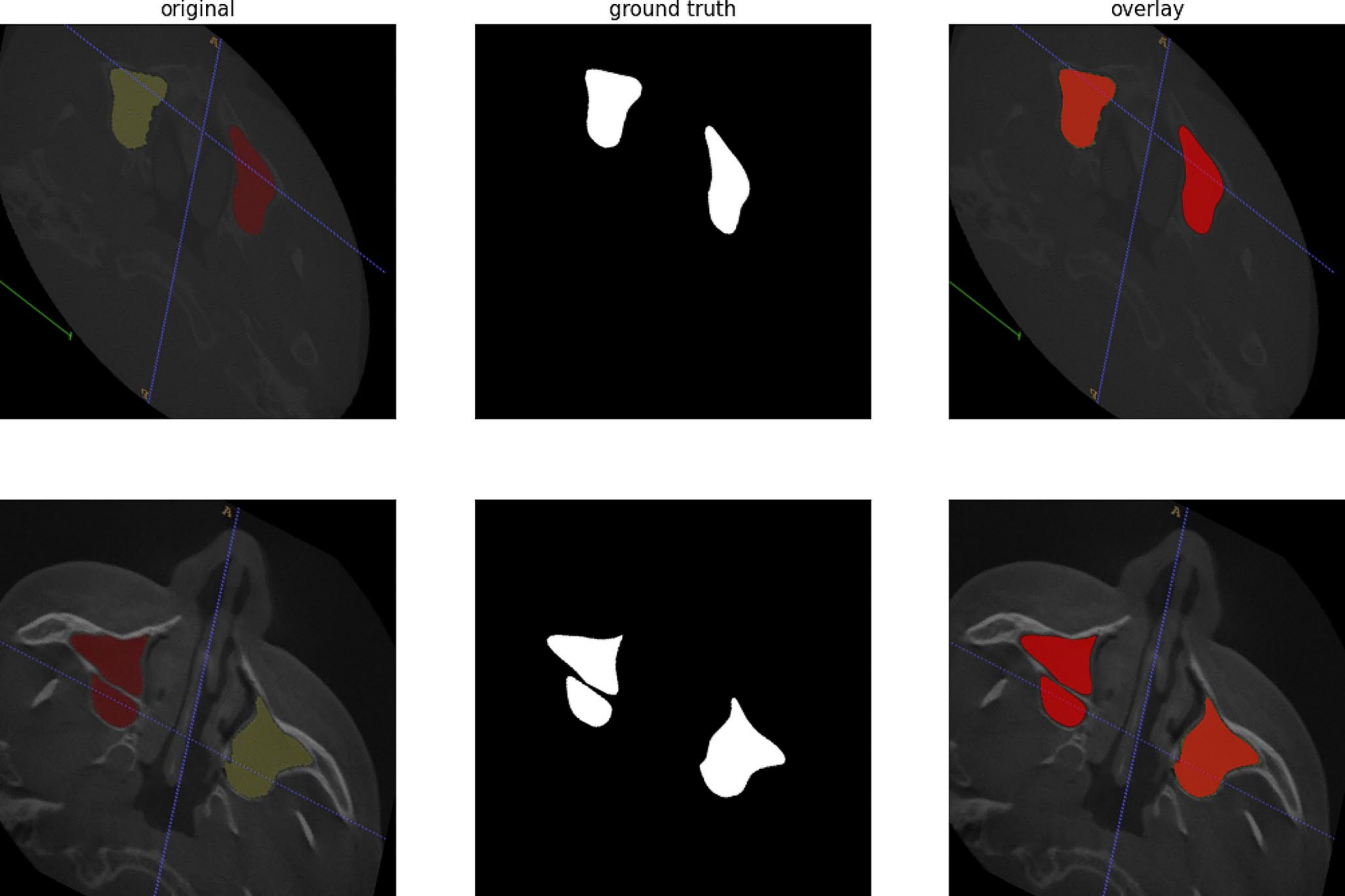
Augmented images examples

The training and testing data of the model are set to 50–50%. Distributing training and testing data equally allows the model to accurately evaluate its performance on both types of data. The fact that the model performs well in both data sets can help increase its generalization ability. Dividing the data set equally can help the model learn the general characteristics of the data set better. If a class is overrepresented (for example, if it makes up 80% of the training data), the model may overfit that class. However, a 50–50% split can help the model generalize better by ensuring it sees enough examples from each class. A balanced data set can increase the model’s ability to distinguish each class. If one class is much more represented than others, the model can identify this class more easily than others. However, an evenly distributed data set can help the model learn each class equally. Balanced training and validation data can help the model perform more reliably on real-world data. The performance of the model on validation data will better reflect the overall performance of the model. Since increasing the number of data will enable segmentation models to learn better, augmentation was applied to each image. Augmented image examples are given in Fig. 5. Data augmentation parameters, descriptions and values are shown in Table 3. These parameters were applied to random images to increase the number of images in the dataset.

**Table 3:** Data augmentation parameters

Parameters	Descriptions	Value
Batch size	Specifies the exhaustion of data sources to be used in each training solution	2
Rotation range	Specifies that the random rotation angle will be between −15 and +15 degrees	15
Width shift range	Specifies the amount of random horizontal scrolling, in percentage	0.05
Height shift range	Specifies the amount of random vertical scrolling, in percentage	0.05
Shear range	Specifies the cutting range in degrees. Shear indicates the amount of strain of an object	50
Zoom range	Specifies the arbitrary zoom range. This specifies the degree to which the image will be zoomed in an arbitrary manner	0.2
Horizontal flip	Specifies that images can be flipped horizontally	True
Vertical flip	Specifies that images can be flipped vertically	True
Fill mode constant	Specifies how to fill in new pixels that may occur during rotation or panning operations. Here it specifies that it will be filled with a fixed color	Constant

**Fig. 6:**
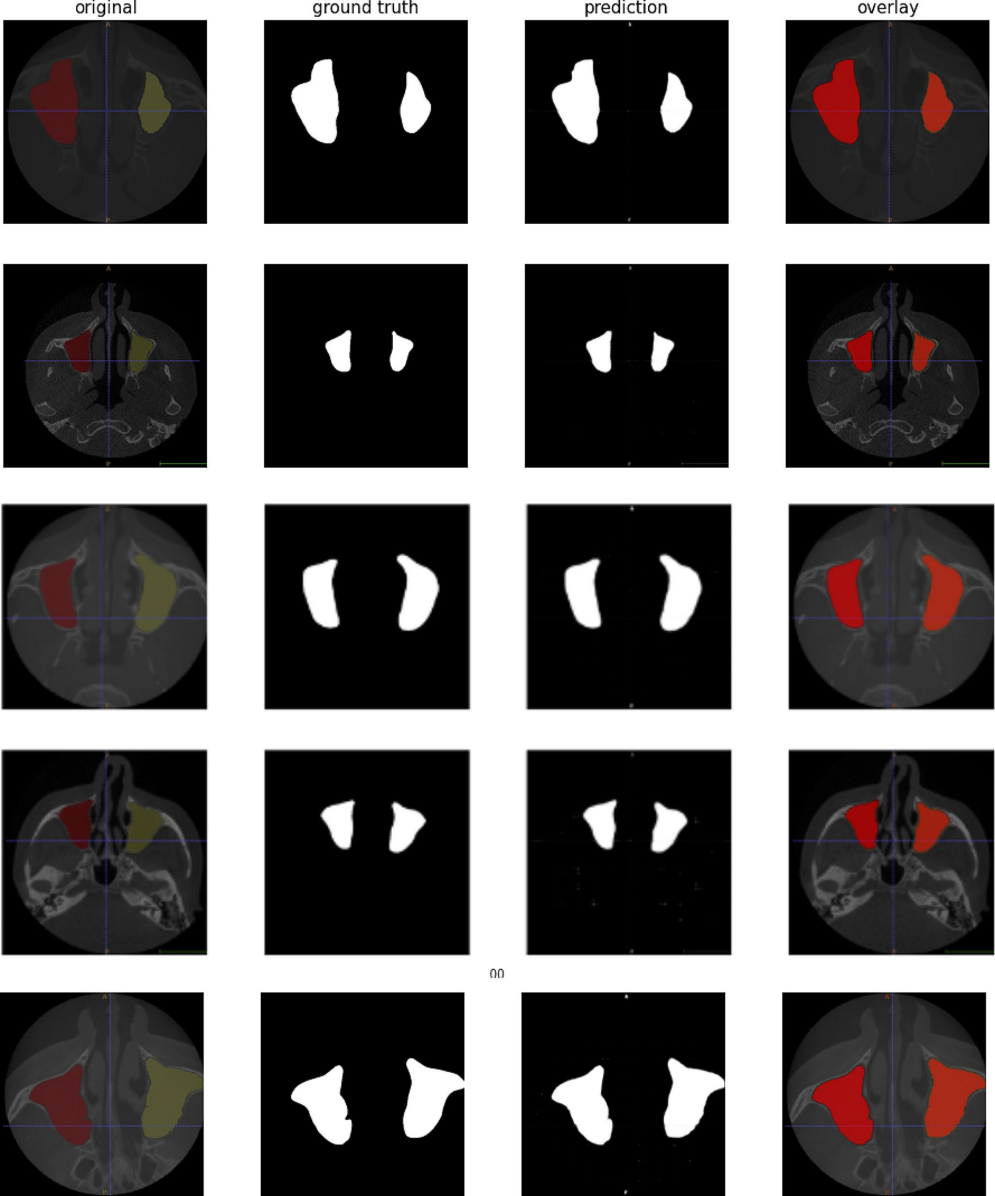
Segmentation examples

In training the U-Net model, the number of epochs was determined as 10 and the number of iterations per epoch was determined as 100. The segmentation images obtained as a result of the training and tests are shown in Fig. 6.

As seen in Fig. 6, training operations were carried out with the original images and masks given as input to the U-Net model. As a result of the training, the test images were given as input to the model and the model was tested. As a result of the tests, the prediction masks in Fig. 6 were obtained. These masks were placed on the original image and the segmentation success of the model was tested. These images are also called overlay. IoU values and F1-Score values of the images were obtained on the overlay images.

As a result of the calculations, the IoU value was found to be 0.9275 and the F1 Score value was 0.9784. IoU measures the agreement between the area predicted by the model and the actual area. It takes a value between 0 and 1, with 1 representing the best fit and 0 representing the worst fit. The IoU value of 0.9275 is high and indicates that the model performs maxillary sinus segmentation accurately. This high IoU value indicates that the model’s predictions are very close to the real data and the segmentation process is successful. Generally, IoU values above 0.5 are considered good, so 0.9275 is a very solid performance indicator. F1 Score is the harmonic mean of precision and recall values. It evaluates whether the model provides balanced accuracy and coverage.

**Fig. 7:**
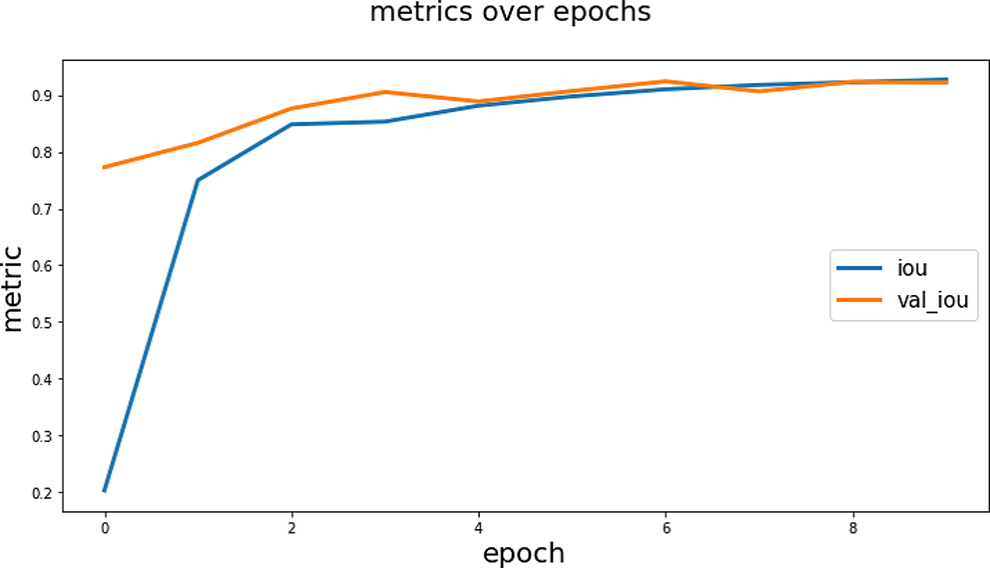
The IoU graph obtained during the training

**Fig. 8:**
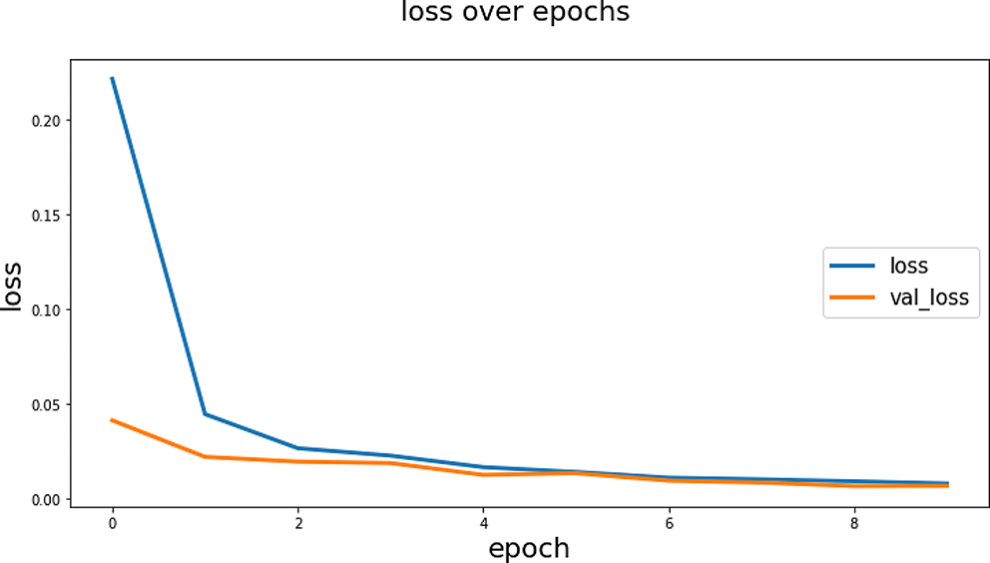
The loss graph obtained during the training

The F1 Score value of 0.9784 is high and indicates that the model has high performance in terms of both precision and sensitivity in maxillary sinus segmentation. This high F1 Score value indicates that the model has low error rates for both true positive predictions and false positives. Since F1 Score is a balanced evaluation metric, it is important to verify that the model works successfully in maxillary sinus segmentation. These high IoU and F1 Score values indicate that the model provides high accuracy and reliability in maxillary sinus segmentation. This increases the usability of the model in clinical applications and can be an important tool for diagnosis and treatment planning. The IoU graph obtained during the training is shown in Fig. 7 and the loss graph is shown in Fig. 8.

According to the graphs in Fig. 7, differences between the training and validation dataset may affect the performance of the model in the training process. For example, being more familiar with the training data and therefore performing better may lead the model to be prone to overfitting. In this case, training IoU values may be high while validation IoU values may be lower. Additionally, different data preprocessing and augmentation processes can create inconsistency between the training and validation dataset. A small validation set may be limited in accurately assessing the model’s performance. The initial state of the model can also affect its responses to the training and validation data sets. A combination of these factors can cause the Validation IoU and Train IoU values to differ at the start of training. However, as the training process progresses, this difference usually decreases, leading to a more balanced performance of the model.

According to Fig. 8, the initial state of the model can be attributed to the model’s better fit to the training data at the beginning of the training process, as the weights have random initial values. This may cause the training loss to be lower than the validation loss. Additionally, differences between the normalization and dropout layers used during training and validation can lead to differences in the initial loss values. For example, dropout layers may be active during training but disabled during validation, causing differences in loss values. Different distributions between training and validation datasets may also contribute to differences in initial loss values. The model may be better able to fit the training data, while the validation dataset may have a more general distribution. Different behavior of the optimization algorithm and parameters during training and validation may also affect the differences in the initial loss values. A combination of these factors can cause the initial validation loss and train loss values to differ. However, as the training process progresses, this difference usually decreases, leading to a more balanced performance of the model.

## Discussion

Paranasal sinuses are air-filled, mucosa-lined cavities located in the maxillofacial region and in communication with the nasal cavity [18]. Maxillary sinuses are found as part of the facial bone and are located just above the maxillary area. The maxillary sinus is the first to develop and the largest paranasal sinus [19]. It has six walls and is a pyramidal space bounded by the alveolar recess, the zygomatic recess, the palatine recess, which lies between the floor of the nasal cavity and the roof of the oral cavity, and the infraorbital recess [20]. The infratemporal fossa is an important deep fascial area located at the periphery of the lateral antral wall [21]. Detection of the maxillary sinuses by image processing is important because it leads to important information for a number of medical and dental applications. The alveolar process forms the inferior wall of the maxillary sinus and is in close relationship with the premolar-molar tooth roots. As a result of this close relationship, periapical lesions arising from premolar-molar tooth roots may cause sinusitis, mucosal thickening, polyps, maxillary retention cysts [22] and oroantral fistula may develop during tooth extraction procedures [23]. Conversely, tooth roots located close to or within the maxillary sinus may also be affected by sinus infections. Maxillary sinus inflammation may develop as a result of bacterial, viral and fungal agents [24]. Sinus lifting procedure may be required due to alveolar bone deficiency during implant placement [25]. Odontogenic cysts and benign tumors cause elevation of the sinus floor, while malignant tumors destroy the sinus floor [26].

Performing manual tasks on a CBCT dataset is difficult and time-consuming. Automated segmentation methods are faster than manual processes and can reduce healthcare professionals’ time and labor. Automated segmentation helps reduce subjective errors and can provide more objective results by reducing differences between experts with different experience levels. The maxillary sinus is connected to neighboring anatomical structures and a multifaceted examination is required. Additionally, analysis results may vary greatly depending on the experts’ knowledge and experience [13, 14]. Deep learning models such as U-Net can be continuously trained and improved, thus providing the basis for future work. Therefore, in this study, the success of automatic segmentation of the maxillary sinus with U-Net was investigated. As a result of the segmentation operations, the IoU value was obtained as 0.9275 and the F1 Score value as 0.9784. It is known that the closer these values are to 1, the more successful the model is. Additionally, when the segmentation images obtained in the study are examined, it can be seen that very successful results are obtained.

Panoramic radiography, which is the most commonly used imaging method in dentistry practice, has limitations in maxillary sinus imaging due to the presence of superimposed anatomical structures such as zygomatic bone and geometric distortion of the images [27]. CBCT stands out in the three-dimensional analysis of the maxillary sinus with its advantages of low cost, high resolution and lower radiation dose compared to CT [28]. Panoramic radiography [11], CT [29] and CBCT [8] have been used in studies on segmentation of the maxillary sinus with different deep learning algorithms.

There are few studies on segmentation of the maxillary sinus in the literature, and algorithms such as UNet [10], 3D UNet [8], 3D nnUNet [30], nn UNet v2 [4], BE-FNet [31], DeepLab [11] and, V-Net [29] are used. Xu et al. [29] proposed a maxillary sinus segmentation network (V-Net) trained on 35 CT datasets and validated its performance using 26 CT testing datasets. The IoU was 90.05 ± 3.26% and the precision was 94.72 ± 2.64%. In anohter study using Panoptic DeepLab, a total of 51 panoramic radiographs were randomly divided into 3 groups: training (*n* = 30), validation (*n* = 11) and testing (*n* = 10) datasets [11]. The IoU was 0.898 for maxillary sinus segmentation. In a study by Yoo et al. [32], comparing the segmentation performances of 2D, 2.5D and 3D networks for the maxillary sinus and maxillary sinus lesions, a total of 67 patients were included. The 2.5D network (U-net + +) showed the highest segmentation performances for the maxillary sinus compared to the 2D and 3D networks. The segmentation performances of Jaccard coefficient, Dice similarity coefficient, precision, and recall by 2.5D network (U-net + +) reached 0.947, 0.973, 0.974, and 0.971. Morgan et al. [8] reported successful results for maxillary sinus automatic segmentation. They collected a dataset of 264 maxillary sinuses from 2 different CBCT devices similar to the present study. A 3D U-Net architecture CNN model was created and contrasted with semi-automatic segmentation in terms of time, accuracy, and consistency. The average time was substantially decreased with automatic segmentation (0.4 min) compared to semi-automatic segmentation (60.8 min). The model precisely delineated the segmented area, achieving a dice similarity coefficient of 98.4%. In a recent study at 2024, Bayrakdar et al. [4] performed maxillary sinus segmentation on 101 CBCT scans using nnU-Net v2. F1 score and IoU values were reported as 0.96 and 0.93, respecively. When comparing our findings with these studies, our proposed method achieved an IoU value of 0.9275 and an F1 Score value of 0.9784, demonstrating successful performance in maxillary sinus segmentation.

U-Net is a deep learning model that, unlike other segmentation methods, provides successful results especially in medical image segmentation. U-Net, which has an encoder-decoder architecture, has an “encoder” structure that encodes the input image into small feature maps and a “decoder” structure that converts these feature maps back to the original size. This architecture shows better performance in high-resolution image segmentation. With the skip connections feature, there are skipping connections between the encoder and decoder in U-Net. In this way, the model can obtain better results by taking into account finer details. U-Net can be trained with little data and generally good results can be achieved by training with transfer learning. U-Net is widely preferred in medical image segmentation because it provides high accuracy, especially in areas such as organ detection, tumor recognition, and lesion segmentation. It is also preferred due to its ability to be trained with little data, fast training times and good results.

### Limitations

Analyzing larger numbers of data obtained from different centers can help evaluate the performance of the model. Combining multiple models offers the potential for better results. In addition to healthy bone borders, including and segmenting images showing pathologies occupying the maxillary sinus will provide guidance before surgery.

## Conclusions

It seems that the proposed U-Net model can be used in maxillary sinus segmentation. In this way, model can provide clinical support by assisting physicians in maxillary sinus examination. In future studies, the use of radiological data collected from different health centers and increased datasets will provide a more comprehensive evaluation.

## Data Availability

The data sets can be shared with researchers who wish to conduct studies upon reasonable request.
